# Characterization of Wear Resistance and Corrosion Resistance of Plasma Paste Borided Layers Produced on Pure Titanium

**DOI:** 10.3390/ma17163922

**Published:** 2024-08-07

**Authors:** Piotr Dziarski, Natalia Makuch

**Affiliations:** Institute of Materials Science and Engineering, Poznan University of Technology, Pl. M. Sklodowskiej-Curie 5, 60-965 Poznan, Poland; piotr.dziarski@put.poznan.pl

**Keywords:** boriding, plasma paste boriding, titanium, wear resistance, corrosion resistance, open circuit potential, polarization curve

## Abstract

Commercially pure titanium was plasma paste borided using various temperatures of the process. An increase in the boriding temperature resulted in an increase in the thickness of the borided layer. All the layers produced consisted of an outer compact TiB_2_ zone and an inner TiB zone in the form of whiskers penetrating into the substrate. The presence of hard titanium borides resulted in a significant increase in wear resistance compared to non-borided pure titanium. However, the thickness of the layer produced strongly influenced the wear behavior, in respect of the time required for complete destruction of the layer. Higher wear resistance was characteristic of the TiB_2_ layer due to its compact nature, whereas the specific morphology of TiB whiskers resulted in their lower wear resistance compared to the outer TiB_2_ layer. Plasma paste boriding of pure titanium also had an advantageous effect on corrosion resistance compared to non-borided pure titanium. Simultaneously, due to the higher thickness of TiB_2_ layer, the specimen borided at a higher temperature showed higher corrosion resistance.

## 1. Introduction

Titanium and its alloys are one of the most widely used materials in the light metal group. The low density of titanium alloys and, simultaneously, their high strength allow these materials to be used in the automotive, shipbuilding, petrochemical, and biomedical industries [1,2,3]. An important property of titanium and its alloys is also good corrosion resistance and stability in most corrosive media. The corrosion resistance of these materials is related to the formation of a continuous, stable, and adherent oxide layer on their surface [4,5]. However, pure titanium and some titanium alloys are unfortunately also characterized by poor wear resistance [6]. A suitable wear protection of titanium and its alloys is provided by thermo-chemical treatment, especially nitriding [7,8,9,10], boriding [2,11,12,13], and carburizing [14,15]. It is very important that the improvement of wear resistance is also accompanied by the improvement or at least maintenance of the original corrosion resistance of titanium. For this reason, the boriding process is an interesting surface treatment that provides both wear and corrosion protection.

Many methods of boriding have been developed to obtain improved tribological properties of titanium and titanium alloys. Powder pack boriding of Ti6Al4V alloy in Ekabor II powder at 1100 °C for 2.5 h resulted in the formation of a dual-phase layer [16]. A high hardness of the titanium borides, above 2000 HV, provided an excellent improvement in wear resistance. The calculated relative wear rate of the borided specimen under dry sliding condition was 100 times lower compared to Ti6Al4V alloy without boriding. A two-times reduction in the coefficient of friction was also achieved [16]. In the case of liquid boriding using a borax-containing borate bath, the application of a temperature of 1000 °C for 12 h resulted in the formation of a titanium boride layer with a total thickness of 42 μm [13]. The wear resistance of borided Cp-Ti and TiAl was analyzed by measuring the relative mass loss compared to the non-borided materials. Both borided specimens showed five times lower relative mass loss compared to that of the non-borided specimens. An interesting alternative to conventional diffusion boriding is laser boriding [17]. Three zones characterized the microstructure obtained by this method laser-borided the re-melted zone containing TiB and TiB_2_, the heat affected zone, and the substrate material. The presence of hard titanium borides in the outer zone improved wear resistance compared to commercially pure titanium. An interesting phenomenon was observed when analyzing the surface of the counter-specimen. The tests of laser-borided layers indicated the catastrophic wear of the counter-specimens, whereas, in the case of pure titanium, wear of the counter-specimen was low [17]. The characteristics of both properties, wear and corrosion resistance, of borided titanium are rarely reported in the literature. 

Pack boriding in powder containing borax and boron carbide was used to form titanium borides on the surface of pure titanium [12]. The friction coefficient COF of the non-borided titanium was higher (0.432) compared to the specimen borided at 920 °C for 20 h (0.284) and borided at 880 °C for 20 h (0.353). The differences in COF values of the borided specimens were related to their surface roughness. The surface roughness after boriding at 920 °C was lower than after boriding at 880 °C. Similarly, the calculated relative mass loss indicated a higher wear resistance of the layer produced at the higher temperature. Analysis of potentiodynamic polarization curves indicated higher corrosion resistance of borided specimens compared to non-borided titanium. However, the corrosion potential of the specimen borided at 920 °C was more positive (−0.562 V in NaCl solution and −0.429 V in H_2_SO_4_ solution) than that of the sample borided at 880 °C (−0.897 V in NaCl solution and −0.438 V in H_2_SO_4_ solution). This can be explained by the continuous, compact, and thick outer TiB_2_ layer formed on the surface of the specimen borided at higher temperature [12]. Powder pack boriding at a high temperature of 1100 °C has also been used to form a hard borides layer on the surface of TB2 alloy [18]. The mass loss after wear tests indicated improved wear resistance, both at room temperature and at 250 °C, after boriding compared to the non-borided titanium alloy. Compared to the non-borided TB2 alloy (*E_corr_* = −0.465 V), the corrosion potential of the borided specimen in a 3.5 wt.% NaCl solution is higher (−0.243 V), and the polarization curve moves towards a positive direction. Similarly, in the case of a H_2_SO_4_ corrosive environment, the borided specimen had a more noble corrosion potential [18]. The advantageous influence of boriding on the corrosion resistance of titanium alloys was also confirmed in other works [19,20]. 

The conventional and most widely used boriding method applied to titanium alloys, the powder pack method [12,16,18,19,20], unfortunately requires high temperatures and a long time duration to produce boride layers of sufficient thickness on Ti-based alloys. Another difficulty when boriding Ti-based alloys is their high reactivity in the presence of oxygen, which results in the formation of a thin oxide layer on the surface. The presence of the oxide layer hinders boron diffusion during conventional powder pack boriding. Both limitations, due to process parameters and the reactive nature of titanium, can be eliminated by using plasma boriding [3]. With the plasma boriding process, a reduction in temperature and process time can be achieved compared to the powder pack method. However, the conventional plasma process variant uses gases such as B_2_H_6_ or BCl_3_, which are expensive, explosive, and toxic. Moreover, corrosion in the vacuum chamber and porosity of the boride layer are other major difficulties characteristic of plasma boriding using gases as a boron source [21]. Therefore, a method of plasma boriding using paste as a boron source (plasma paste boriding) was developed to avoid the problem of toxic gases [22] and additionally to reduce the activation energy of boride layer formation on titanium [23]. Plasma paste boriding using borax as a boron source was an effective method to form a boride layer on pure titanium [11]. The temperature ranged from 750 to 850 °C, and process durations from 3 to 6 h were used. The thickness of the outer TiB_2_ layer and the total thickness of the borided layer increased with increasing temperature and time of the process. The produced layers were characterized by high hardness and wear resistance. The wear resistance of the produced layers, evaluated by the relative mass loss of the borided specimens, depended on the parameters of the plasma paste boriding process. Increasing the temperature and time of the process resulted in an increase in wear resistance [11]. 

In the case of borided titanium alloys, it is very important that the increase in hardness and wear resistance is accompanied by the maintenance of the corrosion resistance of the non-borided base material, or even an increase in its corrosion resistance. Therefore, this paper describes in detail the wear and corrosion resistance of plasma paste borided titanium. Tests were carried out on specimens differing significantly in layer thickness; therefore, it was possible to determine the importance of layer thickness for the obtained properties. Another novel approach is the wear resistance test with the sectioning of the wear diagram into three stages considering the wear of each zone of the borided layer: the outer TiB_2_ layer and the inner TiB whisker zones. This allowed the wear resistance to be compared separately for the TiB_2_ boride and TiB whisker zone. The reasons for the increased wear resistance and corrosion resistance of the borided layer characterized by higher thickness were studied in detail.

## 2. Materials and Methods

Commercially pure titanium, Cp-Ti (Grade 2), was used as the substrate material. Before boriding, the specimens were prepared in the form of a plate with the dimensions of 12 × 12 × 4 mm. 

Plasma paste boriding (PPB) using borax paste (Na_2_B_4_O_7_) was used to form titanium borides on the surface of the samples. Prior to thermo-chemical treatment, the specimens were grounded with SiC abrasive paper to improve the adhesion of the paste to the base material. After cleaning, the top surface of the specimens was coated with a paste containing borax as a source of atomic boron. Such prepared specimens were dried for 24 h at room temperature. Plasma paste boriding processes were carried out using a DC plasma system, described in detail in previous work [11]. The specimens were borided at the temperature of 750 and 850 °C for 6 h in a gas mixture of H_2_ + Ar, with a ratio 1:1, under a constant pressure of 5 ± 0.12 mbar. During the process, the boriding temperature was controlled by changing the input power. A potential difference of 300 ± 5 V and a current density of 0.72 ± 0.03 A provided a temperature of 750 °C. A potential difference of 520 ± 13 V and a current density of 0.78 ± 0.04 A were used to obtain a process temperature of 850 °C. After boriding, the specimens were cooled in a vacuum chamber under a protective atmosphere of argon.

Titanium borides formed by plasma paste boriding were identified by X-ray diffraction (XRD) analysis using a PANalytical EMPYREAN diffractometer (Malvern Panalytical Ltd., Poznan, Poland) with CuKα radiation (λCu = 0.154 nm) and 2θ angles ranging from 30° to 90°. The top surface of the specimen was scanned during XRD analysis, because the borided layer was produced only on it.

A metallographic preparation was carried out to observe the microstructure of borided specimens. First, the specimens were cut perpendicular to the top surface of the specimen on which the borided layer was produced, in order to reveal its cross-section. The prepared specimens were mounted in conductive resin. The metallographic samples were then ground with SiC abrasive papers and polished with an aqueous Al_2_O_3_ suspension. A standard etchant dedicated to titanium and its alloys was used to reveal microstructural details. The etchant consisted of HF, HNO_3_, and H_2_O in a volume ratio of 2:1:47. 

The microstructure was observed using a scanning electron microscope (SEM) Tescan Vega 5135 (TESCAN, Poznan, Poland).

**Figure 1:**
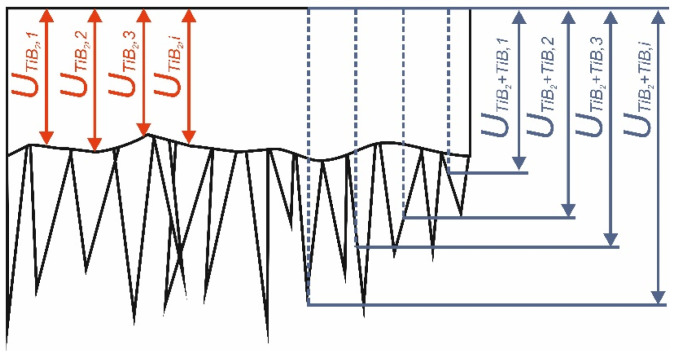
Scheme of the principle of boride layer thickness measurement by Kunst and Schaaber [24] method.

The thickness of boride layers produced at 750 °C for 6 h and at 850 °C for 6 h was calculated as the arithmetic average of 100 measurements. The characteristic morphology of TiB whiskers required a special method for determining their thickness. Therefore, the procedure developed by Kunst and Schaaber [24] was adopted to measure the thickness of the borided layers. A scheme of this method is shown in Figure 1.

The thickness of the continuous TiB_2_ layer (*U_TiB2_*), as well as the total thickness of the layer TiB_2_ + TiB (*U_TiB2+TiB_*), was measured from the top surface. The measurements were performed at regular intervals, i.e., the same distance was used between the separate measurements. Thus, the thickness of the entire layer was measured, taking into account not only the tips of the TiB whiskers. If the whisker was not oriented perpendicular to the top surface, the total layer thickness was completed before the tip of the whisker (e.g., measurement *U_TiB2+TiB,3_*). It was found that considering only the whisker tips would be incorrect.

The thickness of the continuous TiB_2_ layer (*U_TiB2_*) was calculated from equation:

(1) $$U_{{TiB}_{2}}=\sum_{i=1}^{n}\frac{U_{{TiB}_{2},i}}{n}$$

The thickness of the entire layer TiB_2_ + TiB (*U_TiB2+TiB_*) was calculated according to the formula:

(2) $$U_{{TiB}_{2}+TiB}=\sum_{i=1}^{n}\frac{U_{{TiB}_{2}+TiB,i}}{n}$$

where: *n*—number of all measurements (*n* = 100).

Due to the low thickness of plasma paste borided layers produced on pure titanium, hardness was measured using an NHT^3^ nanoindenter (Anton Paar, Poznan, Poland) equipped with a Berkovich diamond tip. The test was carried out under an indentation load of 10 mN, and the hardness was estimated according to the Oliver and Pharr’s method [25]. The detailed experimental procedure for determining the indentation hardness *H_IT_*, as well as the equations used, were described in previous works [11,26,27].

**Figure 2:**
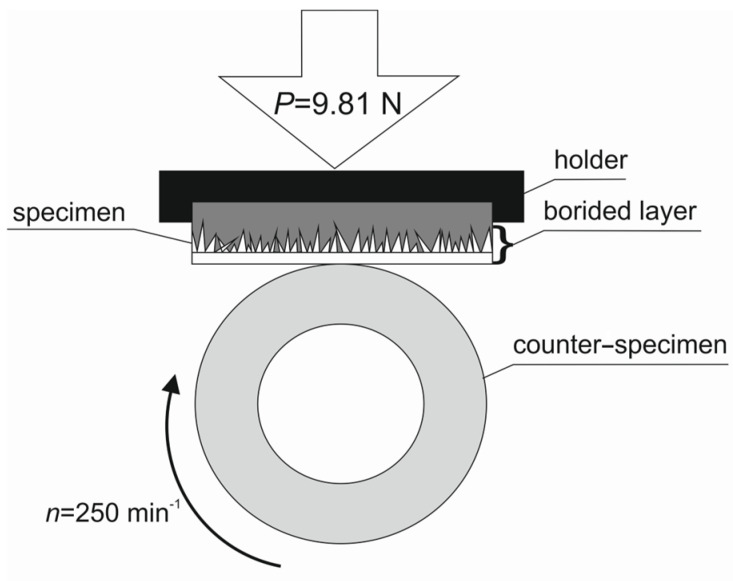
Scheme of the frictional pair used during the wear resistance test.

Wear resistance was evaluated using the frictional pair presented in Figure 2. The specimen in a shape of a plate with dimensions 12 × 12 × 4 mm was stationary during the test and was placed in a holder. A ring-shaped counter-specimen with dimensions Φ20 × 12 mm was rotated at a rate of 250 min^−1^ during the test. The borided layer was produced only on the top surface of the specimen; therefore, it was important to place it properly in the holder in such a way that the borided layer was in contact with the counter-specimen (Figure 2). The counter-specimen was made of quench-hardened and low-temperature tempered 100CrMnSi6-4 bearing steel and had a hardness of 64 HRC. Wear tests were conducted under conditions of dry friction (unlubricated sliding contact) using a load *P* = 1 kgf (9.81 N) for 140 min. Specimen weight was measured every 20 min of the test using a Radwag AS 60/220 R2 analytical balance (Radwag, Poznan, Poland) with a measurement accuracy of ±0.01 mg. Wear resistance was evaluated based on the relative mass loss Δ*m*/*m_i_*, which was calculated for every 20 min of the test. The relative mass loss Δ*m*/*m_i_* is defined as the change in mass of the specimen Δ*m* with respect to the initial weight of the specimen *m_i_* according to the formula:

(3) $$\frac{∆m}{m_{i}}=\frac{m_{i}-m_{t}}{m_{i}}$$

where: *m_i_*—initial mass of specimen before wear test (mg), *m_t_*—mass of specimen measured after a specific test stage (mg).

In order to compare the wear test results obtained for the borided and non-borided specimens with respect to subsequent stages of wear, diagrams representing relative mass loss Δ*m*/*m_i_* as a function of test time *t* were developed. Three stages of wear were identified for both borided specimens: wear of the outer TiB_2_ layer, wear of the inner TiB whisker zone, and wear of the substrate material. The slope coefficient for each stage of the wear test was determined by fitting a linear regression based on the characteristic points for each stage. Points belonging to each stage of the wear test were marked on the diagram of relative mass loss vs. time, and a trend line characterizing the wear stage was added. The square of the correlation coefficient R^2^ for each trend line was also determined. The values of the slope coefficients with the R^2^ parameter are summarized in Table 1.

The 2D profiles of the worn surfaces were determined using a Keyence VHX7000 digital microscope (KEYENCE, Poznan, Poland).

**Figure 3:**
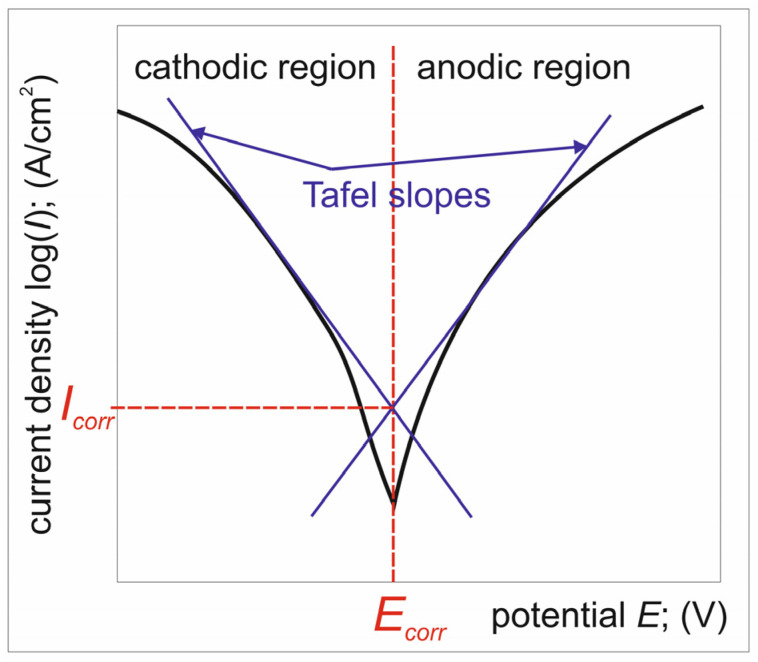
Schematic polarization curve with Tafel’s slope extrapolation principle.

Corrosion resistance was evaluated by two tests: open circuit potential measurements and potentiodynamic anodic polarization. A three-electrode electrochemical cell system was used, with a platinum electrode as the counter-electrode and a saturated calomel electrode (SCE) as the reference electrode. All tests were conducted in a 3.5% NaCl solution using an ATLAS 0531 EU&IA (Atlas Solich, Poznan, Poland) device equipped with Atlas Lab v 2.24 software. The open circuit potential was measured for 7200 s. In the case of potentiodynamic anodic polarization tests, the specimens were polarized in the anodic direction from a potential of −2 V to 2 V, with a potential change rate of 0.5 mV/s. The recorded *E*-log (*I*) polarization curves were analyzed based on the Tafel extrapolation technique [28,29]. Interpretation of the potentiodynamic polarization test using Tafel slope extrapolation enables the determination of two main corrosion parameters: the corrosion potential *E_corr_* and the corrosion current density *I_corr_*. As shown in Figure 3, the polarization curve can be sectioned into two regions: cathodic and anodic. Therefore, based on the Tafel method, it is possible to determine the corrosion parameters (*E_corr_* and *I_corr_*) from a single polarization curve. The Tafel extrapolation technique takes into account the linear parts of the anodic and cathodic curves to determine the corrosion point and corresponding corrosion parameters (Figure 3).

Some authors [30,31] have proposed a simple equation for calculating the corrosion rate *CR* based on the corrosion current density *I_corr_* obtained from the polarization curve. The corrosion rate of the tested specimens was calculated according to the following equation:

(4) $$CR=209\cdot I_{corr}\frac{m}{\rho }$$

where: *m*—mass of the specimen (g), *ρ*—density of the specimen (g/cm^3^).

## 3. Results and Discussion

### 3.1. Microstructure

**Figure 4:**
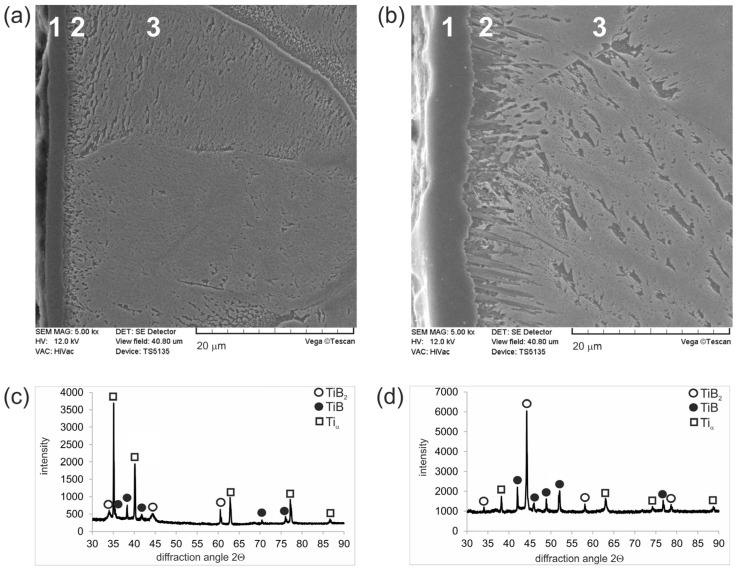
Microstructure (**a**) and diffraction patterns (**c**) of plasma paste borided layer produced on pure titanium at a temperature of 750 °C for 6 h and microstructure (**b**) and diffraction patterns (**d**) of plasma paste borided layer produced on pure titanium at a temperature of 850 °C for 6 h. 1—continuous TiB_2_ layer, 2—TiB whiskers zone, 3—substrate material.

SE images of the plasma paste borided pure titanium were presented in Figure 4. Despite the difference in process temperature, both layers clearly consisted of an outer continuous TiB_2_ boride zone (1) and a TiB whisker zone (2). The presence of both titanium borides was confirmed by XRD phase analysis. Due to the low thickness of the layer produced at 750 °C for 6 h (Figure 4a), numerous peaks corresponding to Ti_α_ were identified (Figure 4c). The morphology of the outer TiB_2_ layer was independent of the process temperature used, but an increase in the boriding temperature resulted in an increase in the thickness of this zone from 2.42 µm for a process temperature of 750 °C to 4.14 µm for a process temperature of 850 °C. In the case of TiB whiskers, the temperature of the process strongly influenced their depth of penetration. A lower boriding temperature resulted in short whiskers with similar penetration depths (Figure 4a), whereas in the case of higher process temperature, the penetration depth of some whiskers into the substrate material exceeded even 10 µm (Figure 4b). The total thickness of the borided layers (TiB_2_ + TiB) was calculated according to the procedure developed by Kunst and Schaaber [24]; therefore, this value differed significantly from the highest penetration depth of the TiB whisker tips. The total thickness of plasma paste borided layers produced on pure titanium also depended on the process temperature. The average total thickness of the layer formed at 750 °C was 3.61 µm, while the layer formed at 850 °C had a total thickness of 7.30 µm.

### 3.2. Indentation Hardness

**Figure 5:**
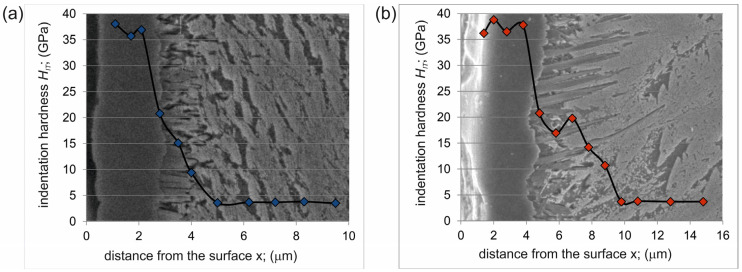
Indentation hardness profile of plasma paste borided layer produced on pure titanium at a temperature of 750 °C for 6 h (**a**) and at a temperature of 850 °C for 6 h (**b**).

The indentation hardness *H_IT_* was measured as a function of the distance from the surface. Measurements were carried out in three zones: the outer continuous TiB_2_ layer, the TiB whisker zone and the substrate material. The obtained hardness profiles for plasma paste borided specimens were presented in Figure 5. In both specimens, borided at 750 °C (Figure 5a) and at 850 °C (Figure 5b), the maximum hardness was obtained in the continuous TiB_2_ layer (35 ÷ 38 GPa). The characteristic morphology of the TiB borides caused the hardness measured in this zone to range from 10 GPa to 20.5 GPa. Such a fluctuation of values requires explanation. The TiB boride had a shape of long and thin whiskers. Therefore, during the hardness tests, only some measurements were made only in this phase. In this case, the higher hardness was obtained and it was characteristic for TiB titanium boride. However, the second zone of the plasma paste borided layer also contained substrate material between the TiB whiskers. Therefore, some of the measurements, especially in the bottom of this zone, were carried out at a boundary between TiB whisker and Ti_α_. A similar situation was described for hardness measured by the Vickers method [11,13].

### 3.3. Wear Resistance

**Figure 6:**
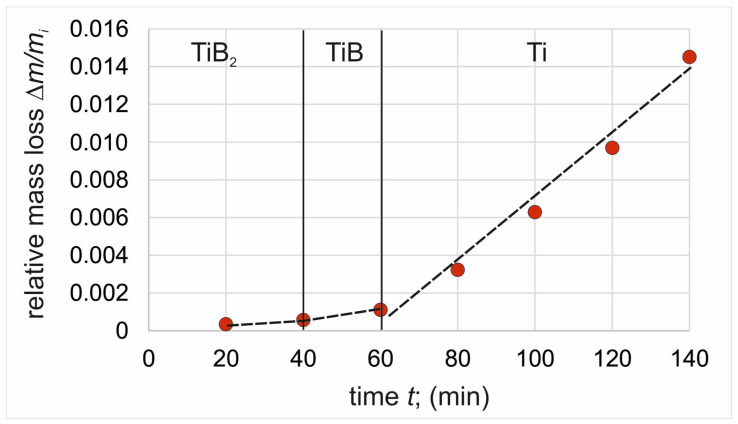
Relative mass loss of plasma paste borided titanium at a temperature of 750 °C for 6 h.

**Figure 7:**
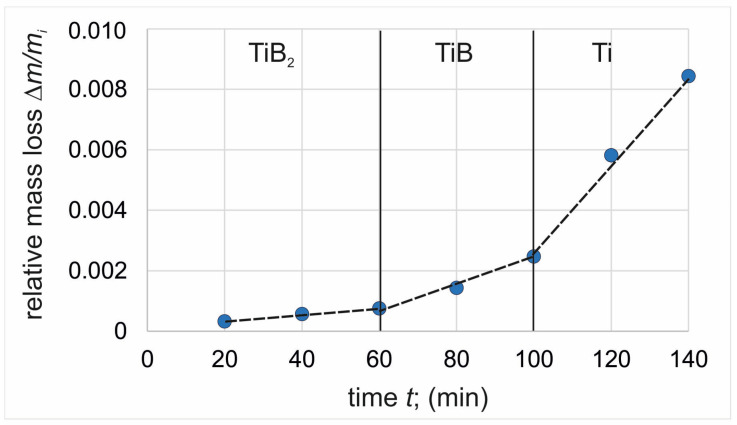
Relative mass loss of plasma paste borided titanium at a temperature of 850 °C for 6 h.

**Figure 8:**
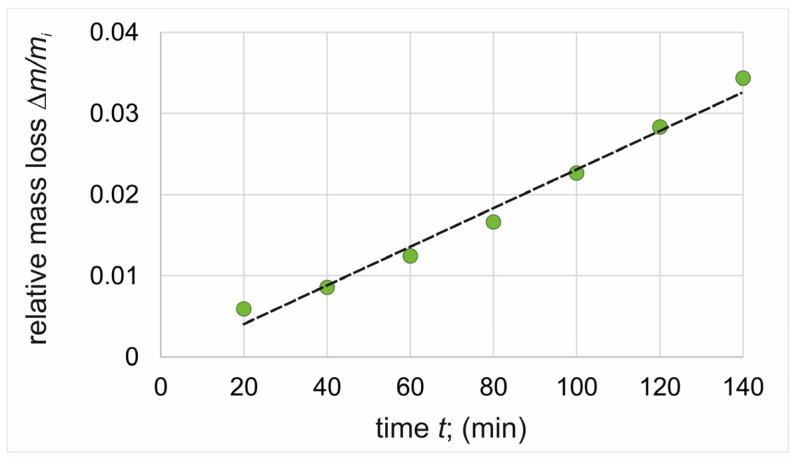
Relative mass loss of non-borided pure titanium.

The thickness of the plasma paste borided layer produced on pure titanium affected the wear behavior of the specimen. In order to compare the two layers differing in thickness, wear tests were conducted with control of mass loss every 20 min of the test. Thus, it was possible to observe the different stages of wear corresponding to the wear of each of the three zones of the borided samples: the continuous TiB_2_ layer, the TiB whiskers zone, and the substrate material. The relative mass loss of the specimens’ plasma paste borided at a temperature of 750 °C and 850 °C were presented in Figure 6 and Figure 7, respectively. For comparison, the wear resistance of non-borided pure titanium was also examined (Figure 8). 

In the case of plasma paste borided specimens, the relative mass loss results were classified into three stages based on the value of mass loss. The first stage corresponded to the wear of the outer compact TiB_2_ layer, whereas the second stage described the wear behavior of the TiB whiskers zone. The last zone corresponded to the wear resistance of the substrate material. In order to easily compare these zones, the slope coefficient of the trend line for each zone was determined and summarized in Table 1. Comparing the slope coefficients calculated for the first zone of both plasma paste borided specimens, it can be concluded that the continuous TiB_2_ boride layer shows similar wear resistance regardless of the process temperature used (0.000013 min^−1^ for specimen borided at 750 °C and 0.000011 min^−1^ for specimen borided at 850 °C). However, it should be noted that the wear time required to completely destroy this zone was longer for the specimen borided at 850 °C (Figure 7). This was an obvious effect of the thickness of the TiB_2_, which was higher for higher boriding temperature. A higher difference between the slope calculated for the borided specimens was observed for the TiB whisker zone. In the case of the specimen borided at 750 °C, the slope coefficient was 0.000064 min^−1^, whereas for the higher boriding temperature it was 0.000051 min^−1^. The reasons for the differences obtained were the characteristic morphology of TiB boride and its depth of penetration into the substrate. The lower boriding temperature resulted in a relatively uniform, but simultaneously significantly lower average depth of the TiB zone (Figure 4a) compared to the layer produced at 850 °C (Figure 4b). Therefore, the time required to completely destroy this zone was longer for the specimen borided at 850 °C (Figure 7).

The effect of TiB whisker tip depth on relative mass loss and slope coefficient was also observed for the third zone (substrate material). In the case of a lower boriding temperature, the slope coefficient calculated for the Ti zone was 0.000202 min^−1^ and was more similar to the value obtained for the non-borided specimen (0.000231 min^−1^). The average depth of TiB whiskers produced by plasma paste boriding at 850 °C was 7.30 µm; however, the penetration depth of some TiB whiskers reached up to 10 µm (Figure 4b). For this reason, the slope coefficient calculated for the Ti zone of the specimen borided at a higher temperature was significantly lower (0.000169 min^−1^). The reason for this situation was the partial presence of TiB whisker tips in the substrate material at the beginning of the third stage of wear, which will be further explained by evaluating the 2D profiles of the wear tracks after the various stages of the test.

**Figure 9:**
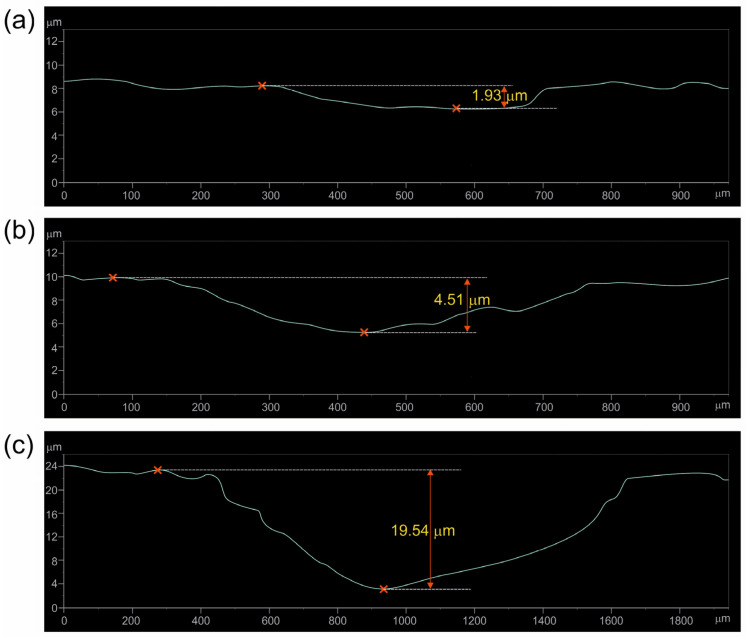
Two-dimensional profile with the measured highest depth of the worn track recorded after 40 min. (**a**), 60 min. (**b**), and 140 min. (**c**) of the wear resistance test performed for the specimen borided at a temperature of 750 °C for 6 h.

**Figure 10:**
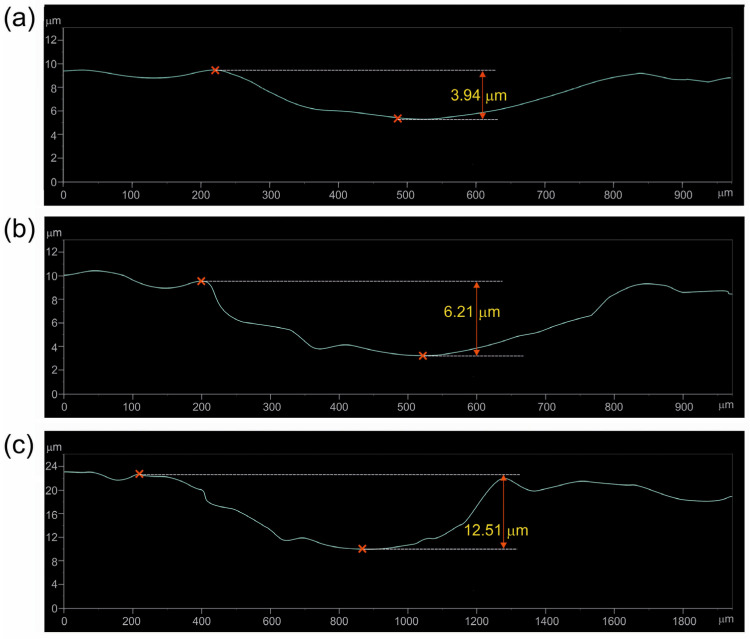
Two-dimensional profile with the measured highest depth of the worn track recorded after 60 min. (**a**), 100 min. (**b**), and 140 min. (**c**) of the wear resistance test performed for the specimen borided at a temperature of 850 °C for 6 h.

To confirm the validity of sectioning the diagrams in Figure 6 and Figure 7 into three stages, the 2D profile of the worn tracks formed on the specimens after a given stage of the test was determined, and the depth of the worn track was calculated. In the case of specimen borided at 750 °C, the profiles were recorded after 40 min. (Figure 9a), 60 min. (Figure 9b), and 140 min. (Figure 9c) of the wear test. For the specimen borided at 850 °C, the profiles were recorded after 60 min. (Figure 10a), 100 min. (Figure 10b), and 140 min. (Figure 10c) of the wear test. The measured depths of the worn tracks after the first stage of wear were compared to the thickness of the continuous TiB layer (*U_TiB2_*), whereas the depths of the worn tracks after the second stage of wear were compared to the total thickness of the layer (*U_TiB2+TiB_*). The comparison is presented in Table 2. 

During the wear test of the specimen borided at 750 °C, the end of the first wear stage was accompanied by a worn track depth of 1.93 µm (Figure 9a). Such depth was close to the average thickness of the TiB_2_ layer measured for this specimen (*U_TiB2_* = 2.42 µm). After 60 min. of the test, the depth of the worn track was 4.51 µm. Considering the average thickness of the entire borided layer measured by the Kunst and Schaaber method, the depth of the worn track (Figure 9b) was much higher (*U_TiB2+TiB_* = 3.61 µm). Obviously, the penetration depth of the TiB whisker tips was higher than the average thickness of the layer, therefore it was assumed that the TiB whiskers zone was completely worn out after 60 min of the wear test. The final depth of the worn track was 19.54 µm and exceeded five times the total thickness of the borided layer produced at 750 °C. Thus, based on the analysis of the 2D profiles of the worn tracks, it can be concluded that the sectioning of the wear diagram into three zones was appropriate and in accordance with the microstructure. 

During the wear test of the specimen borided at 850 °C, the end of the first wear stage was accompanied by a worn track depth of 3.94 µm, obtained after 60 min. of the test (Figure 10a). Such a depth was close to the average thickness of the TiB_2_ layer measured for this specimen (4.14 µm). After 100 min. of the test, the depth of the worn track was 6.21 µm (Figure 10b). This value was slightly lower than the average total thickness of the borided layer (*U_TiB2+TiB_* = 7.30 µm). The final depth of the worn track was 12.51 µm (Figure 10c) and exceeded the total thickness of the borided layer produced at 850 °C. Thus, based on the analysis of the 2D profiles of the worn tracks, it can be concluded that the sectioning of the wear diagram into three zones was appropriate and in accordance with the microstructure. 

Considering the mass loss in relation to the time Δ*m*/*t* (Table 3) required to wear each zone, it was found that the TiB_2_ zone showed the highest resistance to wear by friction. The reason for this was the continuous nature of this layer. The higher resistance of the TiB_2_ zone was also influenced by its high indentation hardness of 35 ÷ 38 GPa (Figure 5). 

In contrast, the specific morphology of the TiB whiskers resulted in a simultaneous mass loss of both the whiskers and the substrate material present between them when testing for wear resistance, hence the greater relative mass loss of this zone. An important piece of general information is that plasma paste boriding of titanium results in an increase in wear resistance compared to the non-borided titanium. Considering both zones of the borided layer, it should be noted that the continuous nature of the outer TiB_2_ layer provided an approximately fifteen-times increase in wear resistance (Table 3). Due to the specific morphology of the TiB boride, the wear resistance of this zone was lower than that of the outer compact TiB_2_ layer. The reason for the lower wear resistance of TiB whiskers zone was the presence of the substrate material between the whiskers. However, it should be noted that the wear resistance of the TiB zone was still five times higher than that of the non-borided titanium (Table 3).

It is very difficult to compare the results obtained in the present study with literature data due to differences in the methodology of conducting wear tests. However, some general information can be concluded. A comparison of the results of wear resistance tests obtained for borided layers produced on titanium alloys by different methods is shown in Table 4. In general, in all cases considered, boriding of titanium or its alloy resulted in a significant increase in wear resistance compared to the non-borided materials. 

The results of wear tests performed for Cp-Ti and Ti6Al4V borided by liquid method [13] proved an increased wear resistance for both borided materials. The calculated relative mass loss was about five times lower in comparison to the non-borided specimen. Relative wear rate (RWR) and friction coefficient (COF) measured for Ti6Al4V after powder boriding at 1100 °C for 2.5h indicated a higher wear resistance for this specimen compared to the non-borided alloy [16]. Unfortunately, there is no information in the literature data presented in [13,16,18] on the effect of the thickness of the borided layer on its tribological properties. Some information on the importance of boriding temperature for wear resistance was presented in the paper [12]. In the case of powder boriding of Cp-Ti [12], the friction coefficient COF of the specimen borided at 920 °C for 20 h (0.284) was lower compared to the specimen borided at 880 °C for 20 h (0.353). The reason for this difference was the lower surface roughness after boriding at 920 °C. Similarly, the calculated relative mass loss Δm/m_i_ indicated the higher wear resistance of a layer produced at higher temperature. The specimen borided at 920 °C was characterized by almost three times lower relative mass loss compared to the specimen borided at 880 °C [12]. The difference in the values of relative mass loss of Cp-Ti borided at different temperatures (880 °C and 920 °C) was explained by the surface characteristics of both layers. The layer produced at 880 °C was porous with the presence of cracks and higher roughness (*R_a_* = 1.217 µm), whereas the layer produced at 920 °C was characterized by lower roughness (*R_a_* = 0.921 µm) and a more compact nature. It was also found that although almost two times higher depth of the inner TiB whisker zone measured for the layer which was produced at 880 °C, the wear resistance of this specimen was significantly lower compared to the specimen borided at 920 °C. This indicates that the thickness and surface characteristic of the outer TiB_2_ layer determine the wear resistance of borided Cp-Ti [12]. The role of TiB_2_ layer thickness for the wear resistance of plasma paste borided Cp-Ti was demonstrated in paper [11]. 

Generally, the significant improvement in wear resistance after plasma paste boriding is caused by the presence of a TiB_2_ layer on the top surface, which has a very high hardness of 35 ÷ 38 GPa. During unlubricated friction of a pair consisting of two materials differing in hardness, the low wear volume is characteristic for the harder material [32]. For this reason, when the friction pair consisted of borided titanium with an outer layer of TiB_2_ (specimen) and hardened 100CrMnSi6-4 bearing steel (counter-specimen), the wear of the harder material, plasma paste borided titanium, was low. In contrast, when the tested specimen was non-borided titanium, which was characterized by a significantly lower hardness than the counter-specimen, a high mass loss was observed for this specimen. In addition to the significant effect of the hard TiB_2_ outer layer on wear resistance, the presence of long TiB whiskers just below it is also important. The TiB whiskers, which admittedly have a lower hardness than the continuous TiB_2_ layer, are distinguished by their characteristic morphology, which ensures excellent adhesion of the entire layer to the substrate material. Some authors indicate that it is the presence of TiB whiskers that is responsible for the damping effect during friction, and at the same time this zone ensures the absence of delamination and breaking of the entire layer [33]. Some authors reported that the presence of a hard TiB_2_ continuous layer on the surface reduced the friction coefficient compared to the non-borided titanium [32,33]. The non-borided titanium is characterized by low hardness (3.5 GPa), and simultaneously is prone to plastic deformation during the wear test. Moreover, titanium easily smears on the surface during the initial period of the wear test, making it difficult to operate at a relatively high coefficient of friction [33].

### 3.4. Corrosion Resistance

The tendency of borided and non-borided specimens to corrosion was investigated using two tests: open circuit potential measurements and potentiodynamic anodic polarization. The open circuit potential (OCP) is a parameter that indicates the thermodynamic tendency of a tested material to oxidation in a corrosive environment. Open circuit potential is defined as the voltage difference between the working electrode and the reference electrode at zero current. 

**Figure 11:**
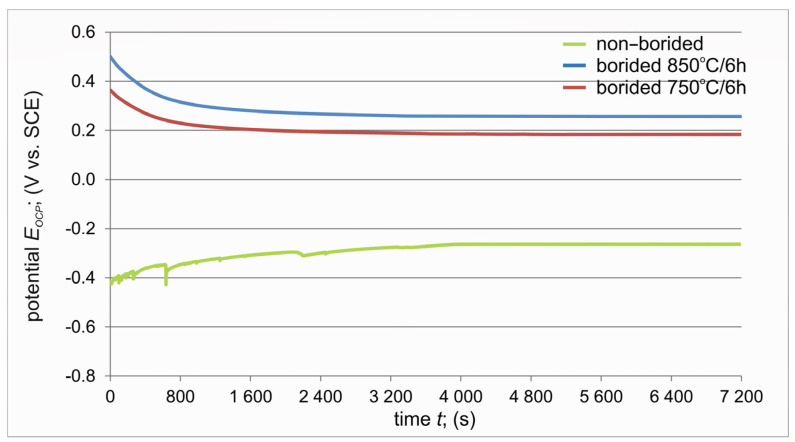
Variation of open circuit potential (OCP) with time for non-borided and plasma paste borided specimens.

Figure 11 shows the *E_OCP_* curves for all specimens immersed in 3.5% NaCl solution. The variations in the open circuit potential were similar for the borided specimens and simultaneously significantly differed from the *E_OCP_* curve recorded for the non-borided titanium. In the case of non-borided pure titanium, the open-circuit potential increased toward the positive potential at the beginning of the test. Such behavior suggests the formation and further thickening of the oxide layer on the metallic surface, improving its ability to protect against corrosion [34,35]. For both borided specimens, the *E_OCP_* decreases with time and gradually stabilizes. Such an open circuit potential profile indicates the absence of passive layer formation on the surface of the borided specimens. Moreover, at the beginning of the test up to 800 s, the sharp decrease in *E_OCP_* of the borided specimens can probably be correlated with the dissolution of the material in the corrosive electrolyte. However, a higher open circuit potential (0.27 V) was obtained for the specimen plasma paste borided at 850 °C for 6 h. Therefore, it was concluded that the borided layer produced at a higher temperature was characterized by a higher corrosion resistance.

**Figure 12:**
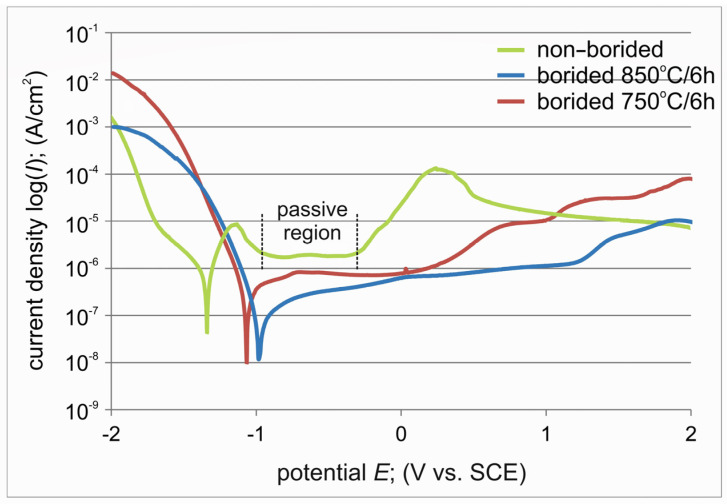
Potentiodynamic polarization curves recorded for non-borided and plasma paste borided specimens.

The potentiodynamic polarization curves recorded for the borided and non-borided specimens are presented in Figure 12. The electrochemical parameters *E_corr_*, *I_corr_*, and *I_pass_* were determined and are compiled in Table 5. The specimen borided at 850 °C was characterized by a higher corrosion potential (*E_corr_* = −0.985 V) than the specimen borided at 750 °C (*E_corr_* = −1.091 V). The non-borided pure titanium demonstrated a lowest corrosion potential of −1.341 V. Both borided specimens were characterized by a lower corrosion current density than the non-borided specimens (Table 5). These two quantities (*E_corr_* and *I_corr_*) indicated that the plasma paste borided specimens show a higher corrosion resistance in 3.5% NaCl solution than the non-borided titanium. However, the layer produced at 850 °C demonstrated a higher resistance to corrosion in 3.5% NaCl solution. The reason for such a situation could be the higher thickness of this layer. Generally, plasma paste boriding caused improvements in corrosion resistance of pure titanium. However, comparing the shape of the potentiodynamic polarization curves (Figure 12), it is clear that only the non-borided specimen is prone to forming a protective passive layer. This was evident in the curve as an area of almost constant current density called passivation current density *I_pass_* (1.96×10^−6^ A/cm^2^). The passivation current density represents the value of the current density recorded for the passive region, in this case in the potential range from −0.988 V to −0.321 V. The characteristic of this region is the almost constant value of the current density, which indicates the presence of the passivation phenomenon of the tested material. Thus, considering the results of the two corrosion tests conducted (open circuit potential measurements and potentiodynamic anodic polarization), it can be summarized that plasma paste boriding improves the corrosion resistance of pure titanium, but the ability to form a protective passive layer is characteristic only of non-borided titanium.

A useful parameter to evaluate corrosion properties is the corrosion rate *CR*. In general, plasma paste boriding provided a reduced corrosion rate in a 3.5% NaCl solution compared to the non-borided specimen. However, producing the layer at a higher temperature provided two times lower corrosion rate (12.522 µm) compared to the layer produced at 750 °C (25.679 µm). The reason for such a situation could be the significantly higher thickness of the layer produced at 850 °C.

The corrosion test results obtained clearly indicate that plasma paste boriding increases the corrosion resistance of titanium. However, an important piece of information is the positive effect of the thickness of the borided layer on the corrosion properties. In all tests, higher corrosion resistance was characteristic of the specimen borided at 850 °C. The increase in corrosion resistance with an increase in boriding temperature was also confirmed in the works [12,19]. The results presented in papers [12,19] are summarized in Table 6 and compared with the present work. In the case of pure titanium borided by the powder method at 880 °C and 920 °C for 20h, the increase in corrosion resistance was accompanied with an increase in boriding temperature, regardless of the corrosive solution used [12]. Comparing the thickness of the layer, it can be concluded that the higher thickness of the outer TiB_2_ layer determined the corrosion parameters. Simultaneously, the lower penetration depth of TiB whiskers in the case of the layer produced at 920 °C did not negatively affect the corrosion resistance of this specimen. The importance of the thickness of the TiB_2_ layer for the corrosion resistance of borided Ti-5Al-2.5Sn alloy was described in paper [19]. It is obvious that the outer TiB_2_ layer is in contact with the corrosion solution during the test; therefore, this phase determines the corrosion resistance of the borided specimen. Corrosion of TiB_2_ boride in 3.5% NaCl aqueous solution proceeds according to the following reactions [19,20]:

(5) $${TiB}_{2}+3H_{2}O\to {Ti}^{4+}+B_{2}O_{3}+6H^{+}$$

(6) $${Ti}^{4+}+2H_{2}O\to TiO_{2}+4H^{+}$$

(7) $$TiO_{2}+2{Cl}^{-}\to TiO_{2}{Cl}_{2}$$

These reactions occur independent of the thickness of the TiB_2_ layer. For this reason, the importance of the TiB_2_ layer thickness for corrosion resistance should be explained by other factors. Corrosion processes accompanied by chloride ions cause the formation of pits and their subsequent propagation deep into the tested material. The outer TiB_2_ layer provides a barrier against corrosion of TiB whiskers and further substrate material. The reason for this is the continuous nature of this layer. In the case of a TiB_2_ layer of low thickness, the pitting that occurs quickly breaks through this zone and corrosion of the TiB whisker zone then occurs. Since this whisker zone is not a continuous boride zone, but there is substrate material between the whiskers, the TiB whisker zone is not an effective barrier against further corrosion. Hence, in all cases considered (Table 6), with decreasing thickness of the outer TiB_2_ layer, the value of the corrosion current density *I_corr_* increases. Corrosion current density represents the amount of material being dissolved into the corrosion solution and thus also determines the corrosion rate. Therefore, the thickness of the outer TiB_2_ layer determines the corrosion resistance and thus also the corrosion rate of the borided sample.

## 4. Conclusions

Plasma paste boriding was used to form dual-phase layers on pure titanium. The outer TiB _2_ layer was continuous and compact, whereas the TiB boride had a morphology of whiskers. The use of two different process temperatures resulted in the formation of layers differing in their thickness. Based on the results obtained from wear resistance tests, as well as corrosion resistance tests, the following conclusions could be formulated:

- Higher wear resistance was characteristic of the TiB_2_ layer due to its compact character and higher hardness (*E_IT_* = 35 ÷ 38 GPa).
- The specific morphology of TiB whiskers and the presence of substrate material between them resulted in their lower wear resistance compared to the outer TiB_2_ layer.
- Considering the time required for the complete wear of the borided layer, it is more advantageous to boride at a higher temperature, which is, obviously, related to the higher thickness of the layer produced.
- The more noble open circuit potential *E_OCP_* was measured for borided specimens.
- The shape of the open circuit potential curve registered for non-borided titanium indicated the tendency of this material to form a protective passive layer.
- The electrochemical parameters (*E_corr_* and *I_corr_*) estimated from the potentiodynamic polarization curves indicated a higher corrosion resistance of both plasma paste borided specimens compared to the non-borided titanium.
- The thickness of the outer TiB_2_ zone determined the corrosion resistance of the borided specimens. In the case of a TiB_2_ layer of low thickness, pitting, which occurs quickly during the test, breaks through this zone easily, and corrosion of the TiB whisker zone occurs. The higher thickness of the TiB_2_ zone hindered the penetration of pits into the TiB whisker zone.
- Since the TiB whisker zone is not a continuous zone, but there is substrate material between the whiskers, the TiB whisker zone is not an effective barrier against further corrosion.
- The shape of the potentiodynamic polarization curve indicated that only non-borided titanium had a tendency toward passivation.

## Figures and Tables

**Table 1:** Slope coefficients of the trend line for each zone calculated for borided and non-borided specimens.

Zone	Specimen Borided at 750 °C for 6 h		Specimen Borided at 850 °C for 6 h		Non-Borided Specimen	
	slope	R^2^	slope	R^2^	slope	R^2^
TiB_2_	0.000013 min^−1^	1	0.000011 min^−1^	0.993	-	
TiB	0.000064 min^−1^	1	0.000051 min^−1^	0.993	-	
Ti (substrate)	0.000202 min^−1^	0.978	0.000169 min^−1^	0.995	0.000231 min^−1^	0.985

**Table 2:** Comparison of measured depths of the worn tracks after first and second stage of wear to the thickness of borided layer.

Wear Stage		Specimen Borided at 750 °C for 6 h	Specimen Borided at 850 °C for 6 h
First	Depth of the worn tracks (µm)	1.93	3.94
	Thickness of TiB_2_ layer *U_TiB2_* (µm)	2.42	4.14
Second	Depth of the worn tracks (µm)	4.51	6.21
	Total thickness of layer TiB_2_ + TiB *U_TiB2+TiB_* (µm)	3.61	7.30

**Table 3:** Mass loss in relation to the time Δ*m*/*t* calculated for borided and non-borided specimens.

Zone	Borided Specimen at 750 °C for 6 h	Borided Specimen at 850°C for 6 h	Non-Borided Specimen
TiB_2_	0.0712 mg/min	0.0617 mg/min	-
TiB	0.2233 mg/min	0.2101 mg/min	-
Ti (substrate)	0.8832 mg/min	0.7285 mg/min	1.1204 mg/min

**Table 4:** Comparison of the wear properties of borided layer produced on titanium alloys using various methods.

Ref.	Substrate Material/Boriding Method	Boriding Parameters	TiB_2_ Layer Thickness (µm)	Total Layer Thickness (µm)	Results of Wear Test	Conditions of Wear Test
[11]	Cp-Ti/plasma paste boriding	non-borided 800 °C/3 h 800 °C/6 h 850 °C/3 h 850 °C/6 h	- 2.44 3.51 3.32 4.04	- 3.97 5.11 5.46 7.30	Δm/m_i_ = 0.000965 Δm/m_i_ = 0.000175 Δm/m_i_ = 0.000087 Δm/m_i_ = 0.000123 Δm/m_i_ = 0.000105	Load: 19.6 N; Counter-specimen: low-temperature tempered 100CrMnSi6-4 steel Test duration: 30 min.
[12]	Cp-Ti/powder boriding	non-borided 880 °C/20 h 920 °C/20 h	- 5 10	- 70 40	COF = 0.432; Δm/m_i_ = 0.00048 COF = 0.353; Δm/m_i_ = 0.00032 COF = 0.284; Δm/m_i_ = 0.00011	Load: 10 N; Counter-specimen: Al_2_O_3_ Test duration: 30 min.
[13]	Cp-Ti/liquid boriding	non-borided 1000 °C/12 h	- 9.4	- 42.4	Δm/m_i_ = 0.00396 Δm/m_i_ = 0.00074	Load: 9.81 N; Counter-specimen: low-temperature tempered 100CrMnSi6-4 steel Test duration: 60 min.
[13]	Ti6Al4V/liquid boriding	non-borided 1000 °C/12 h	- 7.8	- 25.7	Δm/m_i_ = 0.00381 Δm/m_i_ = 0.00073	Load: 9.81 N; Counter-specimen: low-temperature tempered 100CrMnSi6-4 steel Test duration: 30 min.
[16]	Ti6Al4V/powder boriding	non-borided 1100 °C/2.5 h	- 10	- 60	COF = 0.772; RWR = 100% COF = 0.381; RWR = 1%	Load: 12 N Counter-specimen: sapphire ball Test duration: 150 min.
[18]	TB2/ powder boriding	non-borided 1100 °C/20 h	- 7.01	- 21.84	Δm = 1.52 mg; Δm/m_i_ = 0.00034 Δm = 0.61 mg; Δm/m_i_ = 0.00014	Load: 10 N; Counter-specimen: Al_2_O_3_ Test duration: 30 min.
[this work]	Cp-Ti/ plasma paste boriding	non-borided 750 °C/6 h 750 °C/6 h 850 °C/6 h 850 °C/6 h	- 2.4 2.4 4.1 4.1	- 3.6 3.6 7.3 7.3	Δm/t_i_ = 1.1204 mg/min. TiB_2_: Δm/t= 0.0712 mg/min. TiB: Δm/t= 0.2233 mg/min. TiB_2_: Δm/t= 0.0617 mg/min. TiB: Δm/t = 0.2101 mg/min.	Load: 9.81 N; Counter-specimen: low-temperature tempered 100CrMnSi6-4 steel Test duration: 140 min.

**Table 5:** Electrochemical parameters estimated from polarization curves recorded for borided and non-borided specimens.

Parameter	Borided Specimen at 750 °C for 6 h	Borided Specimen at 850 °C for 6 h	Non-Borided Specimen
*E_corr_* (V)	−1.091	−0.985	−1.341
*I_corr_* (A/cm^2^)	1.56 × 10^−7^	7.78 × 10^−8^	8.73 × 10^−7^
*I_pass_* (A/cm^2^)	-	-	1.96 × 10^−6^
*CR* (µm/year)	25.679	12.522	138.450

**Table 6:** Comparison of the effect of borided layer thickness on the corrosion parameters of borided titanium alloys.

Ref.	Substrate Material/Boriding Method	Boriding Parameters	TiB_2_ Layer Thickness (µm)	Total Layer Thickness (µm)	Corrosive Solution	*E_corr_* (V)	*I_corr_* (A/cm^2^)
[12]	Cp-Ti/powder boriding	non-borided 880 °C/20 h 920 °C/20 h non-borided 880 °C/20 h 920 °C/20 h	- 5 10 - 5 10	- 70 40 - 70 40	3.5% NaCl 3.5% NaCl 3.5% NaCl 5% H_2_SO_4_ 5% H_2_SO_4_ 5% H_2_SO_4_	−1.229 −0.897 −0.562 −0.468 −0.438 −0.429	1.414 × 10^−6^ 0.933 × 10^−6^ 0.465 × 10^−11^ 1.101 × 10^−6^ 0.802 × 10^−6^ 0.157 × 10^−6^
[19]	Ti-5Al-2.5Sn/ powder boriding	non-borided 975 °C/12 h 1000 °C/12 h 1025 °C/12 h 1050 °C/12 h 1075 °C/12 h non-borided 975 °C/12 h 1000 °C/12 h 1025 °C/12 h 1050 °C/12 h 1075 °C/12 h	- 13 15 16 16 17 - 13 15 16 16 17	- 22 27 33 43 46 - 22 27 33 43 46	3.5% NaCl 3.5% NaCl 3.5% NaCl 3.5% NaCl 3.5% NaCl 3.5% NaCl 5% H_2_SO_4_ 5% H_2_SO_4_ 5% H_2_SO_4_ 5% H_2_SO_4_ 5% H_2_SO_4_ 5% H_2_SO_4_	−0.655 −0.566 −0.533 −0.494 −0.424 −0.813 −0.757 −0.757 −0.666 −0.590 −0.506 −0.489	0.231 × 10^−8^ 0.129 × 10^−8^ 0.071 × 10^−8^ 0.059 × 10^−8^ 0.044 × 10^−8^ 0.032 × 10^−8^ 0.370 × 10^−5^ 0.135 × 10^−5^ 0.228 × 10^−6^ 0.079 × 10^−6^ 0.044 × 10^−6^ 0.028 × 10^−8^
[this work]	Cp-Ti/ plasma paste boriding	non-borided 750 °C/6 h 850 °C/6 h	- 2.4 4.1	- 3.6 7.3	3.5% NaCl 3.5% NaCl 3.5% NaCl	−1.341 −1.091 −0.985	0.773 × 10^−6^ 0.156 × 10^−6^ 0.778 × 10^−7^

## Data Availability

The authors confirm that the data supporting the findings of this study is available within the article.
